# Early Life Factors Associated with Preschool Wheezing in Preterm Infants

**DOI:** 10.3390/children8090732

**Published:** 2021-08-26

**Authors:** Ying-Lun Hsu, Hsin-Chun Huang, Ting-Yu Su, I-Lun Chen

**Affiliations:** 1 Department of Pediatrics, Kaohsiung Chang Gung Memorial Hospital, Kaohsiung 833, Taiwan; lun0831@hotmail.com (Y.-L.H.); hhuang@cgmh.org.tw (H.-C.H.); teneec8c8c8@hotmail.com (T.-Y.S.); 2 School of Traditional Chinese Medicine, College of Medicine, Chang Gung University, Linkou 333, Taiwan

**Keywords:** asthma, premature infants, risk

## Abstract

Advanced neonatal care has increased the survival of neonates born prematurely, and prematurity is a well-known risk factor for asthma/wheezing disorders. Thus, this prospective study aimed to determine the early life factors associated with preschool wheezing in premature neonates. Preterm neonates born between 2012 and 2017 were recruited, excluding those with bacterial infection within 7 days of life, maternal sepsis, and maternal chorioamnionitis. Birth and admission history, comorbidities, and maternal history were documented. Respiratory problems were followed-up at the neonatal outpatient department. Patients were divided into wheezing and non-wheezing groups. Data were analyzed using the Mann–Whitney test and Fisher’s exact test, and multivariable logistic regression was used to define the risk factors of preschool wheezing/asthma. A total of 125 preterm infants were enrolled, including 19 in the wheezing group and 106 in the non-wheezing group. Patients in the wheezing group had longer duration of intubation (*p* = 0.025), higher rates for exclusive breast milk feeding (*p* = 0.012), and higher re-hospitalization rates for respiratory tract infections (*p* < 0.001), especially for respiratory syncytial virus (RSV) bronchiolitis (*p* = 0.045). The incidence of allergic rhinitis was also higher in the wheezing group (*p* = 0.005). After multivariable logistic regression, allergic rhinitis and re-hospitalization for respiratory tract infections were two significant risk factors for preschool wheezing/asthma in premature neonates. Close follow-up of premature infants at high risk for asthma susceptibility is recommended.

## 1. Introduction

Symptoms of wheezing occurring in children before age 3 years, even transient symptoms, are found to lead to a persistent reduction in lung function by age 6 years [1], and some of these children eventually develop asthma [2]. Recent advances in neonatal intensive care have greatly increased the survival rate of extremely preterm infants. The greater survival, in turn, leads to higher frequencies of lung consequences, especially among those with bronchopulmonary dysplasia (BPD), a major concern for the long-term care of premature infants. Preterm infants have higher rates of early and persistent wheezing compared with full-term infants [3,4].

Asthma affects about 15% of school-age children in Taiwan, and its prevalence among school-age children has been increasing in recent years with contributing factors such as air quality, especially PM_2.5_ [5], increasing parental atopy [6], and changed dietary pattern [7]. The pathophysiology of asthma in children born preterm may be different from that of full-term children. Lower gestational age (GA), lower birth body weight (BBW), and greater infant weight gain are presumed risk factors for asthma/wheezing in preterm births [8,9]. Preexisting genetic and environmental factors such as maternal infection, multiple pregnancy, tobacco use resulting in premature delivery, and immature lung development and immune system with susceptibility to recurrent pulmonary infections, as well as postnatal lung injuries from barotrauma of the ventilator and oxygen toxicity, all may contribute to impaired lung function in preterm infants [10,11]. Preterm neonates may be born by cesarean section [12], a known asthma risk factor, for multiple pregnancy, fetal distress, or maternal complications. Available evidence suggests that, compared to full-term births, preterm births put children at greater risk of developing respiratory diseases in later life, especially obstructive lung disease with impaired lung function, and as a result, a higher prevalence of asthma and chronic obstructive pulmonary disease [10,11]. Children born preterm are also shown to have a lower incidence of atopy, but without lower incidence of asthma, compared with children born at term [13].

During hospitalization, perinatal infection and nosocomial infection are common diseases in premature neonates that may help to protect children from asthma/wheezing. Early life infection can active Th1 cell responses and lead to the production of anti-inflammatory cytokines [14]. However, early use of antibiotics is known for its association with the development of asthma [15]. Moreover, viral infection, especially respiratory syncytial virus (RSV), is an important risk factor for recurrent wheezing [16]. Thus, palivizumab, the monoclonal antibody, has been used since 2010 based on the Taiwan National Health Insurance (TNHI) policy for preventing severe RSV infection in the high-risk population. Previous study revealed the protective effect of palivizumab on the development of asthma in preterm infants [17]. However, evidence from a multicenter study showed that palivizumab prophylaxis administered to preterm infants lowered the incidence of recurrent wheezing but not that of atopic asthma [18]. This study aimed to analyze the risk factors for preschool wheezing in premature infants who needed respiratory support at birth and the effects of palivizumab.

## 2. Materials and Methods

### 2.1. Patient Population

Premature babies who were born at or less than GA 34 weeks between August 2012 and May 2017 were enrolled in this prospective observational study after obtaining signed informed consent from their parents or guardians. Besides the babies born at Chang Gung Memorial Hospital, those who were born elsewhere but admitted to the neonatal intensive care unit (NICU) at Chang Gung Memorial Hospital were also included. Only neonates who needed respiratory support after birth, including nasal continuous positive airway pressure, nasal intermittent mandatory ventilation, or mechanical ventilation, were included. A total of 186 preterm neonates were recruited initially. Neonates with bacterial infections within the first 7 days of life, maternal sepsis, or maternal clinical chorioamnionitis were excluded. Infants with bacterial infections were diagnosed by clinical symptoms and/or proven culture from biofluids, including blood, urine, or cerebral spinal fluid. Clinical chorioamnionitis was defined as maternal fever, uterine fundal tenderness, purulent or foul amniotic fluid, and bacterial growth in the amniotic fluid culture or placenta. Finally, a total of 125 neonates were included for analysis.

The birth and admission history of the included premature infants, including GA, BBW, APGAR score, duration of intubation, breast milk feeding, frequency of infectious episodes, and comorbidities such as BPD, significant patent ductus arteriosus (sPDA), respiratory distress syndrome (RDS), periventricular leukomalacia (PVL), intraventricular hemorrhage (IVH), retinopathy of prematurity (ROP), and necrotizing enterocolitis (NEC), were documented. Breast milk feeding was defined solely at discharge of the neonate. Information regarding maternal history, including maternal education, colonization of group B *streptococcus* (GBS), use of prenatal steroid or antibiotics, smoking history during pregnancy, and maternal disorders such as preeclampsia, fever, gestational diabetes (GDM), and premature rupture of membrane (PROM) were also recorded. BPD was defined as infants requiring more than 21% oxygen for 28 days after birth [19,20]. sPDA implies that the diameter of the PDA is larger than 2.5 mm [21].

### 2.2. Patient Follow-Up

The qualified patients were divided into wheezing (*n* = 19) and non-wheezing (*n* = 106) groups. The operational definition of wheezing included diagnosis by a pediatrician or was defined by the use of asthma medications, such as inhaled selective β2 agonists and/or inhaled corticosteroids (ICS) for more than twice a year for at least 1 year and/or the use of oral leukotriene modifiers for more than 1 month. Neonatologists followed these patients at the outpatient department (OPD) and/or by phone interviews to determine their status, respiratory symptoms, medication history, and any ongoing or chronic illnesses. All patients were followed for at least 3 years. In addition, the documented frequency of wheezing and diagnosis at re-admissions were obtained from the patients’ medical records. The usage of palivizumab in this study was according to the recommendation by the TNHI policy. Before April 2015, all premature infants younger than one year of age and born at or less than 28 weeks of gestation or born at or less than 35 weeks of gestation with BPD were indicated to receive prophylactic palivizumab 750 mg/kg intramuscularly every month for 6 doses. After April 2015, the indication of receiving palivizumab was extended to all premature infants younger than one year of age who were born at or less than 30 weeks of gestation. The first dose was administered within 3–5 days before discharge.

### 2.3. Statistical Analysis

The Mann–Whitney test and Fisher’s exact test were used to analyze continuous and categorical variables, respectively. Continuous variables with non-normal distribution are presented as medians, and categorical variables are presented as percentage (%). The sample size was 126, which was calculated with alpha 5% and power 80%. Demographic information of mothers and neonates, the comorbidities of premature infants (BPD, RDS, sPDA, PVL, IVH, ROP, and NEC), the frequency of re-admission due to respiratory infection, RSV infection, use of palivizumab, and family and atopic history were compared between the wheezing and non-wheezing groups. Multivariable logistic regression by backward selection was used to compare the most significant risk factors in early life for preschool wheezing on children born prematurely. The effect size was measured by odds ratio with 95% confidence interval. Gestational age, birth weight, and significant variables in Table 1 were selected in multivariable models. Though not significant difference, gestational age and birth weight were known as major risk factors in asthma children who were born preterm. However, the effect of gender in asthma development was not reported specifically in premature infants [22]. Therefore, despite no significant difference, we included gestational age and birth weight but did not include gender. The level of significance was set at *p* < 0.05. All statistical analyses were performed using IBM SPSS Statistics 25.0 software (IBM Corp. Armonk, NY, USA).

## 3. Results

A total of 186 preterm infants were recruited in this study initially. Eighteen neonates died during their first hospitalization. Thirty-six of the survivors were excluded due to maternal infections (maternal clinical chorioamnionitis (*n* = 33); maternal sepsis (*n* = 2), and cytomegalovirus (*n* = 1)). Seven babies were excluded because of bacterial infections within the first 7 days of life, (GBS infection (*n* = 3), pneumonia (*n* = 2), and sepsis (*n* = 2)). Finally, a total of 125 infants were included as the analytic sample in this study.

These 125 infants were divided into the wheezing (*n* = 19) and non-wheezing (*n* = 106) groups, based on our operational definition of wheezing. Table 1 shows the characteristics for the two groups. The median GA was 28.1 weeks and 29.9 weeks in the wheezing and non-wheezing groups, respectively. The median BBW was 1140 g and 1265 g in the wheezing and non-wheezing groups, respectively. Despite the lower GA and BBW noted in the wheezing group, no statistically significant differences were observed between the two groups. A longer intubation period was observed in the wheezing group than in the non-wheezing group (*p* = 0.025). No significant differences were observed in the common comorbidities of prematurity, including BPD, grade of RDS, frequency of infectious episodes, sPDA, PVL, IVH, ROP, and NEC, between the two groups. Although there was a higher exclusive breast-feeding rate in the wheezing group than in the non-wheezing group (*p* = 0.012), there was no significance after multivariable logistic regression (Table 2).

A significantly higher frequency of allergic rhinitis and re-hospitalization due to respiratory tract infections was found in the wheezing group than in the non-wheezing group (*p* = 0.005, <0.001). Additionally, patients with RSV infection history were also more likely to develop wheezing (*p* = 0.045). Six infants were diagnosed with laboratory-confirmed RSV infection, and half of them developed recurrent episodes of wheezing later. After multivariable logistic regression, re-hospitalization due to respiratory tract infections and allergic rhinitis were the two most frequent factors associated with wheezing episodes (Table 2).

## 4. Discussion

The results of this study showed that longer intubation periods and higher frequency of respiratory tract infection, especially RSV infection, were associated with preschool wheezing development. Additionally, allergic rhinitis had a higher incidence in infants with recurrent wheezing. However, family atopic history and exclusive breast milk feeding seemed not to be associated with wheezing episodes in preterm infants. A previous study also revealed that wheezing phenotypes in preterm infants were associated with perinatal and postnatal events, not with atopic mechanisms [23].

RSV is the most common pathogen in infantile bronchiolitis, and many studies have demonstrated its link to the development of asthma/wheezing [24,25]. RSV bronchiolitis that is severe enough to warrant hospitalization increases the risk of wheezing, current asthma, and impaired lung function [26]. Preterm infants with and without BPD both easily developed coughing or wheezing in their first year of life [27]. Palivizumab prophylaxis is known to protect children against RSV infection by decreasing wheezing episodes and hospitalization rates in the first 2 years of life [28]. Palivizumab is an FDA-approved monoclonal antibody that is directed against the RSV fusion protein (F glycoprotein) and is recommended by the American Academy of Pediatrics as prophylaxis for high-risk infants younger than 1 year of age [29]. In Taiwan, RSV infections occur throughout the year and without significant seasonality [30]. Thus, the protocol we follow for palivizumab injection, which is based on the TNHI policy, is continuous six doses monthly starting from 3–5 days before discharge, whether during RSV season or not. The protocol is safe and effective for preventing RSV hospitalization in preterm infants [31].

**Figure 1:**
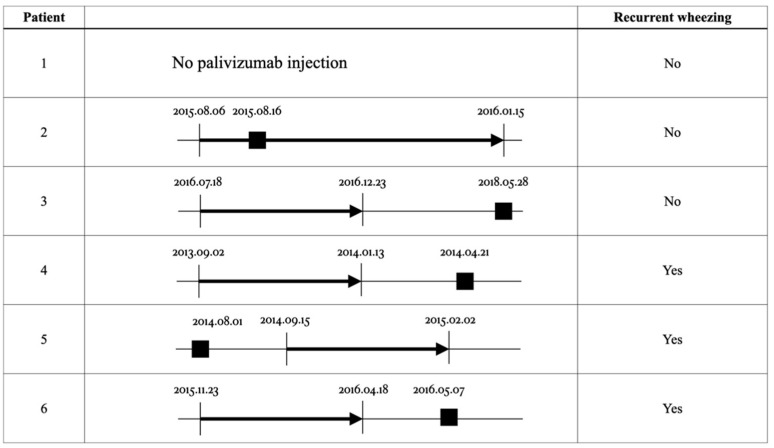
The course of palivizumab prophylaxis and time of RSV infection in six patients. Arrows (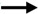) mean the course of palivizumab prophylaxis. Black squares (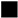) mean RSV infection date. RSV: respiratory syncytial virus.

In the present study, six infants had RSV infection. One of them did not receive palivizumab because it did not conform to the TNHI policy. Another infant had RSV infection during the palivizumab prophylaxis period. An additional four infants had RSV infection, but not within the palivizumab prophylaxis period. Three of these six patients had RSV infection and developed wheezing during their childhood. One had RSV infection before receiving palivizumab prophylaxis, and the other two developed the infection after completing six doses of palivizumab prophylaxis (Figure 1).

Regarding the atopic history of the patients and their families, results of the present study showed no significant difference between the two groups except for allergic rhinitis. The proportion of exclusive breast feeding was higher in the wheezing group than in the non-wheezing group, but without an observed effect on wheezing. A previous study revealed that the incidence of atopy was lower in children who were born preterm than in those born at full term according to the skin prick test, but the incidence of asthma did not decrease [13]. Another study showed that preterm birth was associated with an increased risk of severe asthma and a decreased risk of severe atopic dermatitis [32]. Therefore, the mechanism of asthma development in preterm births may be different from that in full-term infants. It is worth noting that the asthma group had significantly longer duration of intubation (*p* < 0.025). No previous study has demonstrated an association between mechanical ventilation and asthma/wheezing. The mechanism of asthma in premature infants may be explained by multiple factors, such as ventilator-induced injury to the immature lungs or recurrent virus infections. Genetic polymorphism of IL-8 may increase disease severity in infants with RSV infection [33], which may be a predisposing factor for the development of asthma after RSV bronchiolitis.

This study has several limitations that must be mentioned. First, the evaluation of asthma/wheezing disorder in preschool children is difficult due to the lack of objective lung function measurements and biomarkers. Diagnosis usually depends on therapeutic responses. Asthma/wheezing disorder was diagnosed by a pediatrician or was defined by the use of asthma medications twice a year [15]. The wheezing group in the present study did not have significantly lower gestational age and birth weight, compared to those of the non-wheezing group. Small sample size may contribute to these nonsignificant results. Another probable explanation for these known risk factors not reaching statistical significance may be related to the inclusion criteria for this study. Only premature infants born at or less than 34 weeks of gestation and who needed ventilator support were included, not all preterm neonates, which may have led to selection bias. Furthermore, environmental factors such as tobacco smoke, air pollution, molds and fungi, indoor chemicals, and household factors have an important impact on wheezing disorders [34,35]. Although all of our enrolled patients were living in southern Taiwan, large differences and variations in air pollution levels are found, which may influence the degree of wheezing and/or asthma susceptibility. Tobacco smokes of family members living together were not recorded. Household factors as pets, dust mites, and cockroaches are hard to evaluate.

Recurrent wheezing during preschool childhood is associated with poorer lung function and higher airway resistance [36,37], which suggests increased asthma susceptibility. In the present study, longer intubation periods, hospitalization for RSV bronchiolitis, and allergic rhinitis were associated with preschool wheezing. Knowledge of the correlation between early life factors and wheezing disorders may help to guide early intervention among high-risk infants, such as avoiding longer intubation at preterm birth and avoiding RSV infection. Extending the availability of RSV monoclonal antibody through palivizumab injection may be considered for these infants.

## Figures and Tables

**Table 1:** Baseline characteristics between wheezing and non-wheezing groups.

	Wheezing (*n* = 19)	Non-Wheezing (*n* = 106)	*p*-Value
**Birth history**			
Gestational age (wk)	28.1 (25.9–30.9)	29.9 (27.6–31.3)	0.070
Birth weight (g)	1140 (840–1460)	1265 (917.5–1572.5)	0.407
Male/Female	12/7	54/52	0.455
APGAR score at 1 min	5 (3–6)	6 (4.5–7)	0.196
APGAR score at 5 min	8 (6–9)	8 (7–9)	0.244
**Maternal history**			
Mode of delivery(C/N)	9/10	40/66	0.453
Prenatal steroid (%)	84.2	74.0	0.561
Group B streptococcus (%)	0	4.0	1.000
Prenatal antibiotic (%)	84.2	78.6	0.761
Smoking during pregnancy (%)	5.3	4.8	1.000
Preeclampsia (%)	15.8	14.2	0.737
Maternal fever (%)	10.5	5.7	0.350
PROM (%)	36.8	43.4	0.626
Gestational diabetes (%)	5.3	3.8	0.568
College education (%)	35.3	46.5	0.440
**Admission history**			
Intubation duration (day)	41 (0–76)	3.5 (0–10)	0.025
Surfactant administration (%)	63.2	59.4	0.805
Infection frequency	0 (0–2)	0 (0–1)	0.086
Exclusive breast milk feeding (%)	68.4	36.8	0.012
RDS grade	2.0 (1.0–2.5)	2.0 (1.0–2.5)	0.699
Bronchopulmonary dysplasia (%)	84.2	72.6	0.397
Significant PDA (%)	21.0	23.6	1.000
Peri-ventricular leukomalacia (%)	5.3	5.7	1.000
Intraventricular hemorrhage (%)	21.1	20.8	1.000
ROP (%)	31.6	22.6	0.394
Necrotizing enterocolitis (%)	21.1	15.2	0.508
**Follow-up history**			
Re-admission for res. infection	3 (1–5)	0 (0–1)	<0.001
RSV infection (%)	15.8	2.8	0.045
Use of Palivizumab (%)	68.4%	55.7%	0.327
**Atopic History**			
Allergic rhinitis (%)	31.6	6.6	0.005
Atopic dermatitis (%)	10.5	3.8	0.226
Family atopic history (%)	15.8	17.0	1.000

C/N: caesarean section/normal spontaneously delivery; PROM: premature rupture of membranes; RDS: respiratory distress syndrome; PDA: patent ductus arteriosus; res.: respiratory; RSV: respiratory syncytial virus. Continuous variables with non-normal distribution are presented as medians. Categorical variables are presented as percentage (%). Significant difference is defined as *p* < 0.05.

**Table 2:** Multivariable logistic analysis of early life factors between wheezing and non-wheezing groups.

	Model 1	Model 2	Model 3	Model 4	Model 5	Model 6
Gestational age (wk)	0.98 (0.57–1.68)					
Birth weight (g)	1.00 (0.99–1.00)	1.00 (0.99–1.00)				
Intubation duration (days)	1.01 (0.97–1.05)	1.01 (0.97–1.04)	1.01 (0.97–1.04)	1.00 (0.97–1.04)		
Exclusive BM feeding (%)	0.47 (0.11–2.04)	0.46 (0.11–1.95)	0.48 (0.12–1.96)	0.48 (0.12–1.97)	0.47 (0.12–1.91)	
Re-admission for res. infection	3.32 (1.70–6.49) ***	3.31 (1.70–6.45) ***	3.40 (1.82–6.37) ***	3.43 (1.84–6.40) ***	3.55 (2.02–6.24) ***	3.62 (2.01–6.34) ***
RSV infection (%)	0.69 (0.05–9.14)	0.68 (0.05–8.97)	0.74 (0.06–8.76)			
Allergic rhinitis (%)	0.12 (0.02–0.63) *	0.12 (0.02–0.63) *	0.12 (0.02–0.63) *	0.12 (0.02–0.63) *	0.12 (0.02–0.63) *	0.13 (0.02–0.65) *

* *p* < 0.05; *** *p* < 0.001; BM: breast milk; RSV: respiratory syncytial virus. res.: respiratory. Model 1: multivariable logistic analysis with gestational age, birth weight, exclusive breast milk feeding, allergic rhinitis, intubation period, re-admission for respiratory infection, and RSV infection. Model 2: birth weight, exclusive breast milk feeding, allergic rhinitis, intubation period, re-admission for respiratory infection, and RSV infection were analyzed. Model 3: exclusive breast milk feeding, allergic rhinitis, intubation period, re-admission for respiratory infection, and RSV infection. Model 4: exclusive breast milk feeding, allergic rhinitis, intubation period, and re-admission for respiratory infection. Model 5: exclusive breast milk feeding, allergic rhinitis, and re-admission for respiratory infection. Model 6: allergic rhinitis and re-admission for respiratory infection. Data are presented with ORs and 95% CI.

## Data Availability

The data presented in this study are available on request from the corresponding author. The availability of the data is restricted to investigators based in academic institutions.
